# Simultaneous transcatheter valve-in-valve replacement of severely degenerated bioprosthetic aortic and mitral prostheses

**DOI:** 10.1007/s00392-022-02059-2

**Published:** 2022-08-04

**Authors:** Hendrik Wienemann, Victor Mauri, Elmar Kuhn, Stephan Baldus, Matti Adam

**Affiliations:** 1 Clinic III for Internal Medicine, University of Cologne, Faculty of Medicine and University Hospital Cologne, Kerpener Str. 61, 50937 Cologne, Germany; 2 Department of Cardiothoracic Surgery, University of Cologne, Faculty of Medicine and University Hospital Cologne, Kerpener Str. 61, 50937 Cologne, Germany

## Abstract

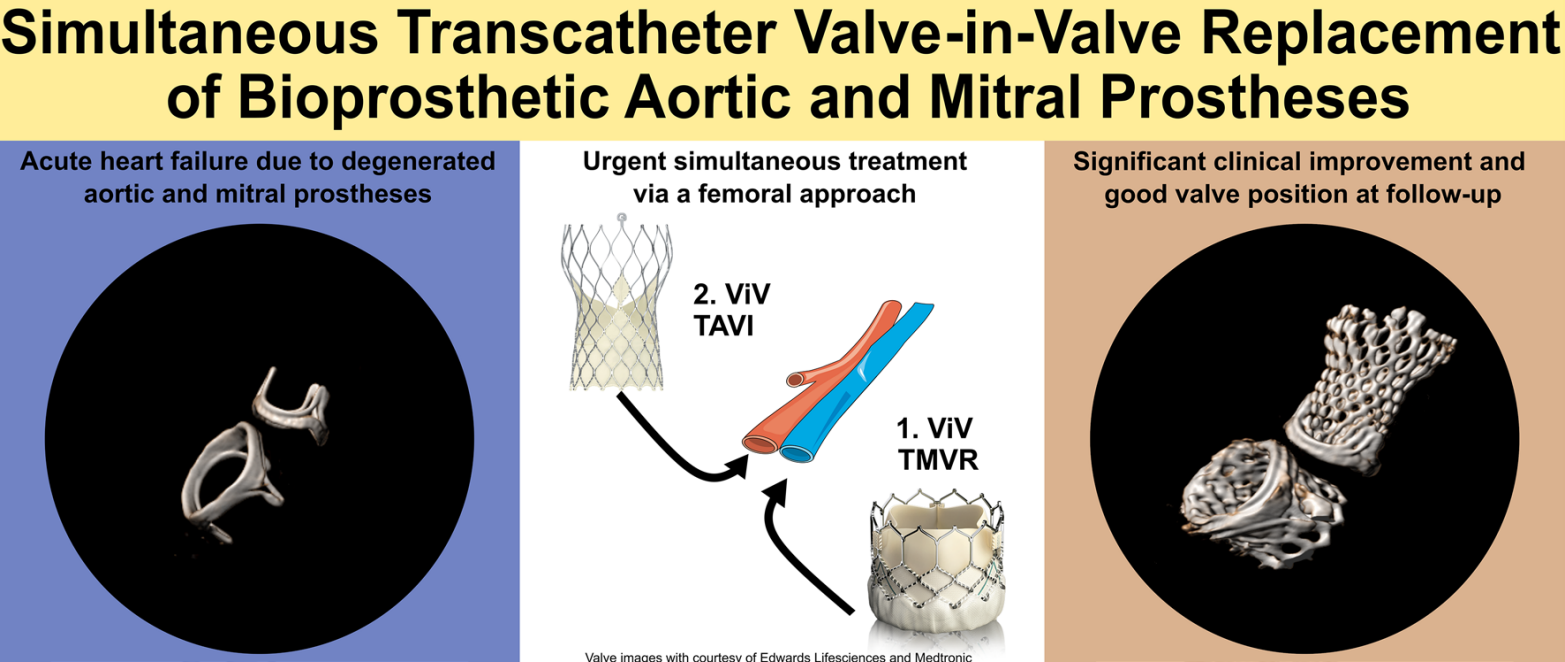

Sirs:

In patients who require surgical heart valve replacement, bioprosthetic heart valves (BHVs) are frequently utilized as the treatment of choice. As life expectancy is constantly growing, valve durability becomes more important and structural valve deterioration needs to be therapeutically addressed. Transcatheter aortic valve-in-valve replacement (ViV-TAVR) is increasingly considered for the treatment of patients with degenerated aortic valve prostheses.

More recently, valve-in-valve transcatheter mitral valve replacement (ViV-TMVR) has emerged as a treatment option for patients with symptomatic severely degenerated bioprosthetic valves with high risk for redo cardiac surgery [1].

Concomitant degeneration of aortic valve (AV) and mitral valve (MV) prostheses might occur more frequently as the population ages. In these patients, progressive structural valve deterioration of both valves may have cumulative effects on circulation and may rapidly cause severe congestive heart failure and left atrial thrombus formation. Currently, a considerable proportion of these patients are not suitable for redo cardiac surgery.

Therefore, urgent simultaneous double valve replacement might be required. While previous investigators have reported simultaneous ViV-TAVR and ViV-TMVR in patients via a transapical access with balloon-expandable valves [2, 3], data on transfemoral access route with the optimal treatment strategy is limited. We report a case of successful transfemoral simultaneous ViV-TAVR and ViV-TMVR in degenerated bioprosthetic valves.

An 85-year-old male patient was presented with symptomatic dyspnea NYHA class IV and unstable angina to our emergency department. Clinical examination revealed signs of low forward cardiac output with pulmonary and peripheral edema. The patient was stabilized by i.v. diuretic therapy and oxygen support. Past medical history comprised atrial fibrillation, hypertension, chronic kidney disease and a history of prior strokes. At the age of 72 concomitant surgical septal myectomy and bioprosthetic mitral (Carpentier-Edwards Perimount 31 mm) and aortic valve (Carpentier-Edwards Perimount 23 mm) replacement for severe mitral regurgitation and aortic stenosis has been performed.

Transthoracic and transesophageal echocardiography (TOE) revealed severe bioprosthetic mitral valve stenosis (valvular orifice area of 0.3 cm^2^) and concurrent severe aortic valve bioprosthetic stenosis (valvular orifice area of 0.96 cm^2^ and low-flow low-gradient stenosis due to the severe impairment of the mitral valve prosthesis). Furthermore, left ventricle (LV) size was within normal limits with an impaired ejection fraction of 33%. On cardiac catheterization, no coronary artery disease was found. Computed tomography (CT) imaging enabled assessment of valve dimensions (Fig. 1). The patient had favorable anatomy with good transfemoral access options and no suturing of the atrial septum during the original surgical intervention. Aorto-mitral angle was steep with 72° and neo LVOT was calculated with 413 mm^2^, yielding a low risk for LVOT obstruction following ViV-TMVR. The preoperative logistic EuroSCORE II for redo surgery in this patient was calculated with 38.8%. Taken together, the patient showed a prohibitively high risk for redo surgical dual valve replacement due to deteriorating clinical status.

**Fig. 1:**
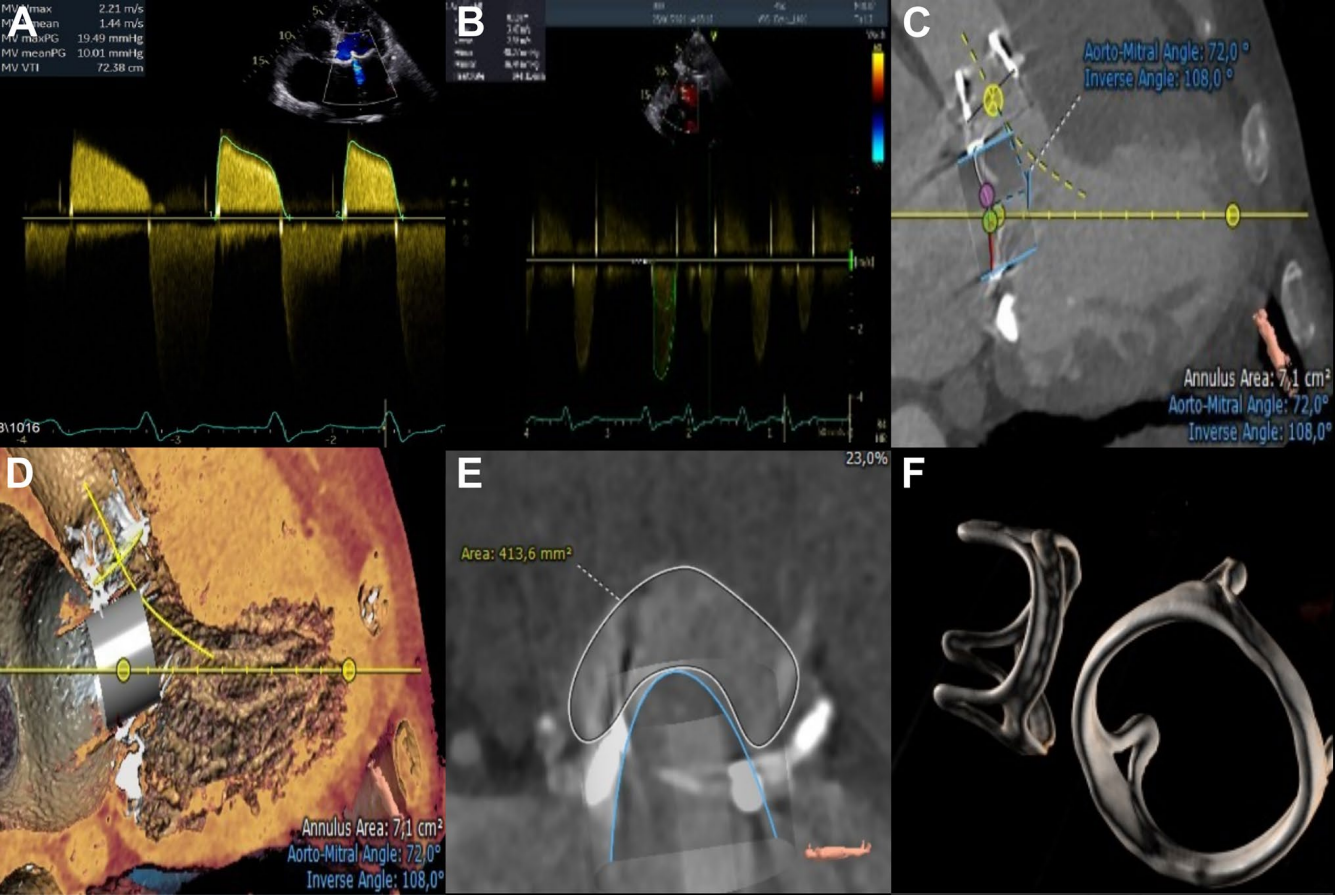
**A** Two-dimensional transthoracic echocardiography showing severe mitral valve stenosis with a mean gradient of 10 mmHg. **B** Baseline aortic valve assessment showing a severe low-flow low-gradient stenosis (valve orifice area, 0.96 cm^2^; mean gradient, 36 mmHg; max gradient, 48 mmHg). **C** Cardiac CT depicting a steep aorto-mitral angle with 72°. **D** CT simulation of the ViV-TMVR deployment showing positioning of the valve and adjacent neo-LVOT. **E** Calculation of neo-LVOT with an area of 413 mm^2^, indicating a low risk for LVOT obstruction after MV implantation. **F** CT displaying the bioprosthetic heart valves in aortic and mitral position

Therefore, a complete percutaneous transfemoral approach was chosen by the heart team. We hypothesized that in this diseased left ventricle, acutely increased LV preload after solitary treatment of mitral valve stenosis would result in marked systolic dysfunction, especially after consideration of the hemodynamically relevant stenotic AV prosthesis. To minimize the risk of acute LV failure, we decided to perform subsequent ViV-TMVR and ViV-TAVR in one procedure.

The 31 mm Carpentier-Edwards Perimount valve (Edwards Lifesciences, Irvine, CA, USA) in mitral position had a tissue annulus diameter of 33.5 mm and a stent diameter of 31 mm. Therefore, the SAPIEN 3-Ultra (29 mm) transcatheter heart valve (S3-Ultra; Edwards Lifesciences, Irvine, CA, USA) was chosen as the most suitable treatment option.

The 23 mm Carpentier-Edwards Perimount valve (Edwards Lifesciences, Irvine, CA, USA) in aortic position had a stent diameter of 22 mm and a true inner diameter of 21 mm. The self-expandable Evolut R (26 mm) valve (Medtronic, Minneapolis, MN, USA) was selected for treatment. Venous access was consequently conducted via a 16F eSheath (Edwards Lifesciences, Irvine, CA, USA) and arterial access was realized with a 14F Sentrant Sheath (Medtronic, Minneapolis, MN, USA) with subsequent in-line sheath deployment of the valve.

Atrial septal puncture was performed with a Brockenbrough needle via a Mullins sheath (Cook Group, Bloomington, IN, USA) from the right femoral vein aiming for an anterior and inferior septal puncture. Due to a prior history of stroke and planned valvuloplasty of the mitral valve prosthesis, a Sentinel cerebral protection device (Boston Scientific, Marlborough, MA, USA) was positioned via the right radial artery. Subsequently, a Lunderquist wire was advanced into the left ventricle and the interatrial atrial septum was dilated using an Osypka VACS II 16 mm balloon (Osypka AG, Rheinfelden, Germany), followed by predilatation of the MV prosthesis utilizing an Osypka VACS II 25 mm balloon (Osypka AG, Rheinfelden, Germany). The S3-Ultra 29 mm was introduced and deployed under rapid ventricular pacing, aiming for a 90% ventricular position and 10% atrial stent position. TOE showed proper position of the S3-Ultra prosthesis with good motion of the leaflets and no signs of paravalvular regurgitation. In addition, the low forward aortic flow with elevated left ventricular filling pressure required intensive hemodynamic support to maintain adequate hemodynamic control. Now, a stiff guidewire (Safari2™, Boston Scientific, Marlborough) was positioned in the LV after passing the AV prosthesis via an AL1 catheter from the arterial side. Entanglement of the wire and the newly implanted S3-Ultra mitral prosthesis was fluoroscopically excluded. Next, a Medtronic Evolut R was implanted in the usual way with fast pacing for valve positioning and release (Fig. 2). X-ray angiograms and TOE revealed no paravalvular leakage and showed adequate distance between valve prostheses without LVOT obstruction. The delivery systems and sheaths were retracted. Hemostasis was achieved by Proglide sutures for arterial access and Z-stitches followed by manual compression at the venous site.

**Fig. 2:**
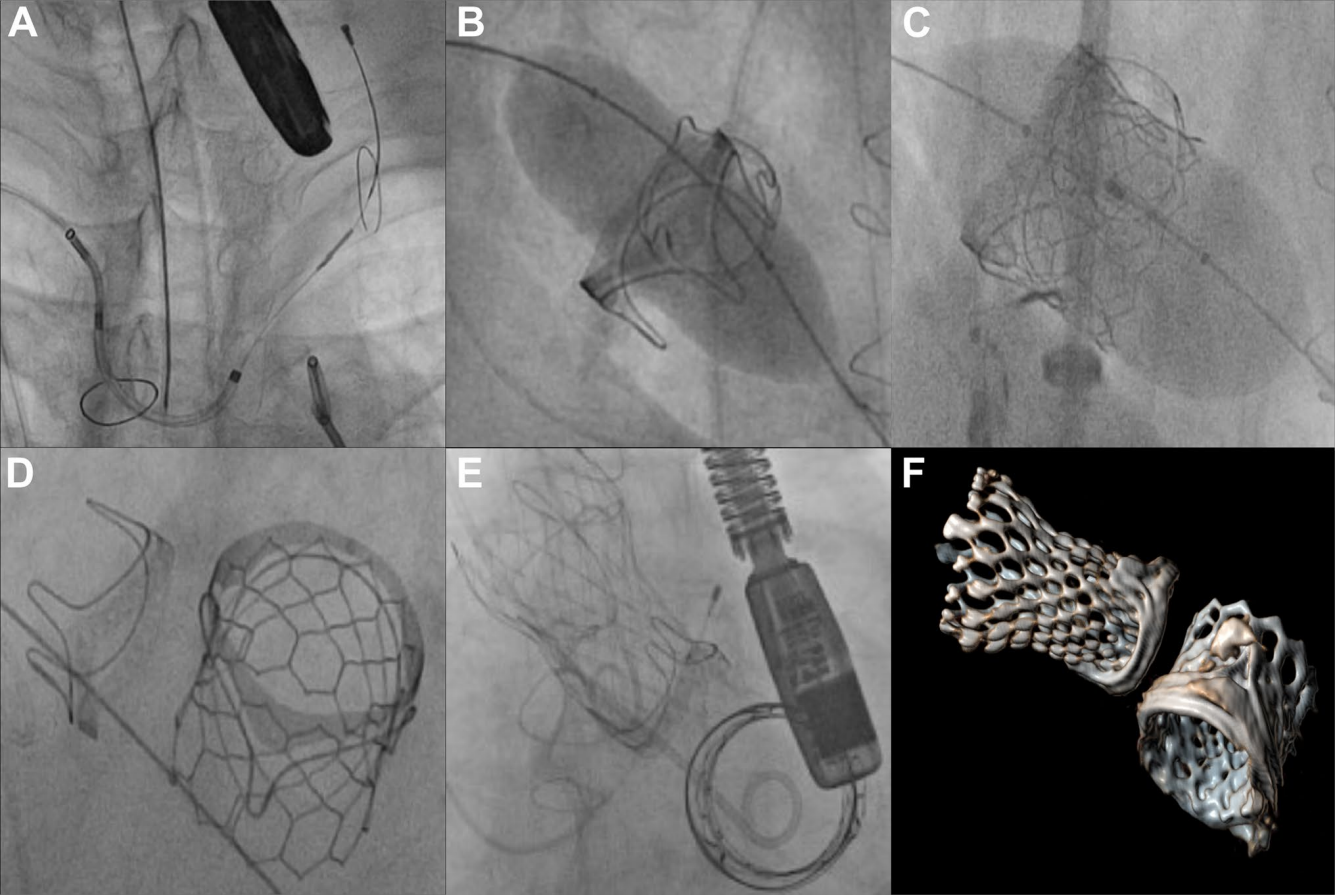
**A** Positioning of a Sentinel cerebral protection device in standard position covering brachiocephalic trunk and left common carotid artery. **B** Predilatation of the prosthetic mitral valve and **C** Implantation of the SAPIEN 3-Ultra 29 mm. **D** Result of ViV replacement in mitral position. **E** Result of ViV replacement in aortic position after implantation of Evolut R 26 mm. **F** Follow-up CT revealed well-seated transcatheter heart valves in aortic and mitral valve position

Postprocedural course was stable and without complications. The patient was discharged on day seven. Follow-up examinations were conducted after 6 weeks, 3 and 6 months and 1 year. The patient reported no relevant dyspnea (NYHA I) and no limitations in activities of daily life. Physical examination revealed no signs of congestive heart failure. Echocardiography and CT demonstrated none/trace paravalvular or transvalvular regurgitation for both valves (Fig. 2.). Follow-up echocardiography revealed a mean mitral valve gradient of 3.5 mmHg and a valve area of 1.4 cm^2^. The implanted ViV-TAVR demonstrated trace paravalvular regurgitation and a mean gradient of 9 mmHg with a valve area of 2.2 cm^2^.

Whereas ViV-TAVR provides a feasible and safe alternative to surgical aortic valve replacement in patients with structural valve degeneration [4], ViV-TMVR procedures show promising early outcome data [5], but need to achieve similar evidence [6]. As single valve procedures get more standardized, especially patients in critical condition might benefit from single shot-double valve interventions in terms of procedural success.

In our case, an anticipated critical clinical state with subsequent volume overload of the left ventricle after ViV-TMVR led to the decision of simultaneous valve implantation, apprehending a flow increase over the AV prosthesis with subsequent high-flow high-gradient stenosis. The risk of an afterload mismatch due to LV deterioration following ViV-TMVR should be considered [7] and was here acutely aggravated by AV prosthesis stenosis. Transcatheter double valve implantation might represent a good bailout option especially in high-risk patients with prior valve replacement.

The first ViV-TAVR was performed in 2007 [8], followed in 2009 by a ViV-TMVR [9] via a transapical access. Next, Lutter et al. reported in 2020 good 6 months follow-up of a patient, who was treated simultaneously with transapical ViV-TMVR and ViV-TAVR implantation [2]. As transapical access is often associated with worse procedural und outcome in transcatheter valve procedures [10], transfemoral access might be considered more desirable. Two case reports described their successful experiences of percutaneous simultaneous double valve replacement in patients with native severe aortic and mitral valve stenosis with balloon-expandable valves [11, 12].

Self-expanding valves often demonstrate superior valve hemodynamics compared to balloon-expandable valves in native aortic valve TAVR [13]. Therefore, their supra-annular design might also provide hemodynamical benefits for patients with small AV prosthetic diameters after concomitant ViV-TAVR.

This case adds to the body of evidence showing feasibility and safety of simultaneous double valve-in-valve procedures with contemporary balloon-expandable and self-expanding TAVRs via a transfemoral approach. To achieve optimal results, meticulous CT-based procedure planning is important.

## References

[1] Simonato M, Whisenant B, Ribeiro HB, Webb JG, Kornowski R, Guerrero M, Wijeysundera H, Søndergaard L, de Backer O, Villablanca P, Rihal C, Eleid M, Kempfert J, Unbehaun A, Erlebach M, Casselman F, Adam M, Montorfano M, Ancona M, Saia F, Ubben T, Meincke F, Napodano M, Codner P, Schofer J, Pelletier M, Cheung A, Shuvy M, Palma JH, Gaia DF, Duncan A, Hildick-Smith D, Veulemans V, Sinning J-M, Arbel Y, Testa L, de Weger A, Eltchaninoff H, Hemery T, Landes U, Tchetche D, Dumonteil N, Rodés-Cabau J, Kim W-K, Spargias K, Kourkoveli P, Ben-Yehuda O, Teles RC, Barbanti M, Fiorina C, Thukkani A, Mackensen GB, Jones N, Presbitero P, Petronio AS, Allali A, Champagnac D, Bleiziffer S, Rudolph T, Iadanza A, Salizzoni S, Agrifoglio M, Nombela-Franco L, Bonaros N, Kass M, Bruschi G, Amabile N, Chhatriwalla A, Messina A, Hirji SA, Andreas M, Welsh R, Schoels W, Hellig F, Windecker S, Stortecky S, Maisano F, Stone GW, Dvir D (2021). Transcatheter mitral valve replacement after surgical repair or replacement: comprehensive midterm evaluation of valve-in-valve and valve-in-ring implantation from the VIVID registry. Circulation.

[2] Lutter G, Salem M, Frank D, Puehler T (2020). One-shot transcatheter double valve replacement: six-month follow-up—A case report. Eur Heart J Case Rep.

[3] Seiffert M, Baldus S, Conradi L, Koschyk D, Schirmer J, Meinertz T, Reichenspurner H, Treede H (2011). Simultaneous transcatheter aortic and mitral valve-in-valve implantation in a patient with degenerated bioprostheses and high surgical risk. Thorac Cardiovasc Surg.

[4] Sá MPBO, van den Eynde J, Simonato M, Cavalcanti LRP, Doulamis IP, Weixler V, Kampaktsis PN, Gallo M, Laforgia PL, Zhigalov K, Ruhparwar A, Weymann A, Pibarot P, Clavel M-A (2021). Valve-in-valve transcatheter aortic valve replacement versus redo surgical aortic valve replacement: an updated meta-analysis. JACC Cardiovasc Interv.

[5] Whisenant B, Kapadia SR, Eleid MF, Kodali SK, McCabe JM, Krishnaswamy A, Morse M, Smalling RW, Reisman M, Mack M, O'Neill WW, Bapat VN, Leon MB, Rihal CS, Makkar RR, Guerrero M (2020). One-year outcomes of mitral valve-in-valve using the sapien 3 transcatheter heart valve. JAMA Cardiol.

[6] Eleid MF, Whisenant BK, Cabalka AK, Williams MR, Nejjari M, Attias D, Fam N, Amoroso N, Foley TA, Pollak PM, Alli OO, Pislaru SV, Said SM, Dearani JA, Rihal CS (2017). Early outcomes of percutaneous transvenous transseptal transcatheter valve implantation in failed bioprosthetic mitral valves, ring annuloplasty, and severe mitral annular calcification. JACC Cardiovasc Interv.

[7] Quintana E, Suri RM, Thalji NM, Daly RC, Dearani JA, Burkhart HM, Li Z, Enriquez-Sarano M, Schaff HV (2014). Left ventricular dysfunction after mitral valve repair—the fallacy of “normal” preoperative myocardial function. J Thorac Cardiovasc Surg.

[8] Wenaweser P, Buellesfeld L, Gerckens U, Grube E (2007). Percutaneous aortic valve replacement for severe aortic regurgitation in degenerated bioprosthesis: the first valve in valve procedure using the core valve revalving system. Cathet Cardiovasc Intervent.

[9] Cheung A, Webb JG, Wong DR, Ye J, Masson J-B, Carere RG, Lichtenstein SV (2009). Transapical transcatheter mitral valve-in-valve implantation in a human. Ann Thorac Surg.

[10] Blackstone EH, Suri RM, Rajeswaran J, Babaliaros V, Douglas PS, Fearon WF, Miller DC, Hahn RT, Kapadia S, Kirtane AJ, Kodali SK, Mack M, Szeto WY, Thourani VH, Tuzcu EM, Williams MR, Akin JJ, Leon MB, Svensson LG (2015). Propensity-matched comparisons of clinical outcomes after transapical or transfemoral transcatheter aortic valve replacement: a placement of aortic transcatheter valves (PARTNER)-I trial substudy. Circulation.

[11] Fanari Z, Mahmaljy H, Nandish S, Goswami NJ (2018). Simultaneous transcatheter transfemoral aortic and transeptal mitral valve replacement using Edward SAPIEN S3. Catheter Cardiovasc Interv.

[12] Bashir M, Sigurdsson G, Horwitz PA, Zahr F (2017). Simultaneous transfemoral aortic and transseptal mitral valve replacement utilising SAPIEN 3 valves in native aortic and mitral valves. Euro Intervention.

[13] Hahn RT, Leipsic J, Douglas PS, Jaber WA, Weissman NJ, Pibarot P, Blanke P, Oh JK (2019). Comprehensive echocardiographic assessment of normal transcatheter valve function. JACC Cardiovasc Imaging.

